# Airway epithelial immunoproteasome subunit LMP7 protects against rhinovirus infection

**DOI:** 10.1038/s41598-022-18807-3

**Published:** 2022-08-25

**Authors:** Kris Genelyn Dimasuay, Niccolette Schaunaman, Bruce Berg, Diana Cervantes, Elke Kruger, Frank L. Heppner, Deborah A. Ferrington, Hong Wei Chu

**Affiliations:** 1 Department of Medicine, National Jewish Health, Denver, CO USA; 2 Institute for Medicine Biochemistry and Molecular Biology, Universitätsmedizin Greifswald, Greifswald, Germany; 3 Department of Neuropathology, Charité-Universitätsmedizin Berlin, Corporate Member of Freie Universität Berlin and Humboldt-Universität zu Berlin, Berlin, Germany; 4 Doheny Eye Institute, University of California Los Angeles, Pasadena, CA USA

**Keywords:** Infection, Inflammation, Innate immunity, Mucosal immunology

## Abstract

Immunoproteasomes (IP) serve as an important modulator of immune responses to pathogens and other pathological factors. LMP7/β5i, one of the IP subunits, plays a critical role in autoimmune diseases by downregulating inflammation. Rhinovirus (RV) infection is a major risk factor in the exacerbations of respiratory inflammatory diseases, but whether LMP7 regulates RV-mediated inflammation in the lung particularly in the airway epithelium, the first line of defense against RV infection, remains unclear. In this study, we determined whether airway epithelial LMP7 promotes the resolution of RV-mediated lung inflammation. Inducible airway epithelial-specific LMP7-deficient (conditional knockout, CKO) mice were generated to reveal the in vivo anti-inflammatory and antiviral functions of LMP7. By using LMP7-deficient primary human airway epithelial cells generated by CRISPR-Cas9, we confirmed that airway epithelial LMP7 decreased pro-inflammatory cytokines and viral load during RV infection. Additionally, airway epithelial LMP7 enhanced the expression of a negative immune regulator A20/TNFAIP3 during viral infection that may contribute to the anti-inflammatory function of LMP7. We also discovered that induction of LMP7 by a low dose of polyinosinic:polycytidylic acid (PI:C) reduced RV-mediated inflammation in our CKO mice infected with RV. Our findings suggest that airway epithelial LMP7 has anti-inflammatory and antiviral functions that is critical to the resolution of RV-mediated lung inflammation. Induction of airway epithelial LMP7 may open a novel avenue for therapeutic intervention against RV infection.

## Introduction

Immunoproteasomes (IP), alternative form of the constitutive proteasome, are generated upon exposures to pro-inflammatory stimuli such as interferon-γ and TNF-α^1^. In non-immune cells (e.g. epithelial cells), IP subunit-containing proteasomes are induced by interferons while IP subunits in some types of immune cells (e.g. T cells) are constitutively expressed. IP also has stronger proteolytic functions than the constitutive proteasome. It has been recognized as an important modulator of both innate and adaptive immune responses against bacterial and viral infections^2^. Studies have shown the role of IP in inflammation and viral infection, which may be independent of its classical function in antigen presentation^3,4^. Briefly, IP was shown to reduce the host susceptibility to infection against *Candida albicans*^5^, *Toxoplasma gondii*^6^, hepatitis B virus^7^, and coxsackievirus B3^8^. By using a global low-molecular mass protein 2 (LMP2, one of the IP subunits) gene knockout mouse model, our group was the first to demonstrate a role of IP in enhancing antiviral function during rhinovirus (RV) infection in vivo^9^. However, our previous work was not able to demonstrate the contribution of each of the IP catalytic subunits (i.e. LMP2/β1i, LMP7/β5i, and MECL-1/β2i) against lung RV infection particularly its role in the airway epithelium, the primary site and first line of defense against RV infection.

Among the three IP catalytic subunits, LMP7 has been found to have a critical role in the incorporation and acceleration of the other subunits during IP assembly^10^. Deficiency in LMP7 impairs the incorporation of the IP subunits LMP2 and MECL-1 into the proteasome core leading to the accumulation of immature proteasome precursors^11,12^. Additionally, mutations in the human PSMB8 gene encoding LMP7 results in proteasome-associated autoinflammatory syndromes^13–15^, which are characterized by severe inflammation^16^. Likewise, mice-deficient in LMP7 showed strong susceptibility to autoimmune encephalomyelitis or pancreatitis^17,18^. Taken together, these studies support an important role of LMP7 in regulating inflammatory responses. However, the function of LMP7 subunit is still unknown in the lung airway epithelium particularly in the context of RV infection. RV is the most common trigger of asthma exacerbations^19^ by increasing airway inflammation and tissue injury. Currently, there is no effective vaccine or therapy for RV infection. To date, there were only two other studies demonstrating a role of IP in lung viral infections including murine gammaherpesvirus-68 (MHV-68)^20^ and porcine reproductive and respiratory syndrome virus (PRRSV)^21^. Similar to what we found with RV^9^, both studies demonstrated that IP is crucial in the activation of antiviral immune response against viral infection in the lung.

Here, we delved deeper into uncovering the role of LMP7 in the resolution of RV-mediated inflammation specifically in the airway epithelium. We hypothesized that airway LMP7 has anti-inflammatory and antiviral functions that contributes to the resolution of inflammation against RV infection by promoting airway epithelial expression/function of A20/TNFAIP3, a negative regulator of NF-κB signaling^22^. We further hypothesized that an appropriate level of LMP7 induction by a low dose of polyinosinic:polycytidylic acid (PI:C) reduces RV-mediated airway inflammation. We utilized airway epithelial-specific LMP7-deficient in vivo and in vitro models to test our hypotheses. A better understanding on the mechanism of the airway epithelium is critical to maintain host innate inflammatory and immune response against invading pathogens like RV, which may open novel avenue for effective therapy.

## Results

### Airway epithelial LMP7 promotes resolution of lung inflammation and viral clearance in RV-infected mice

**Figure 1:**
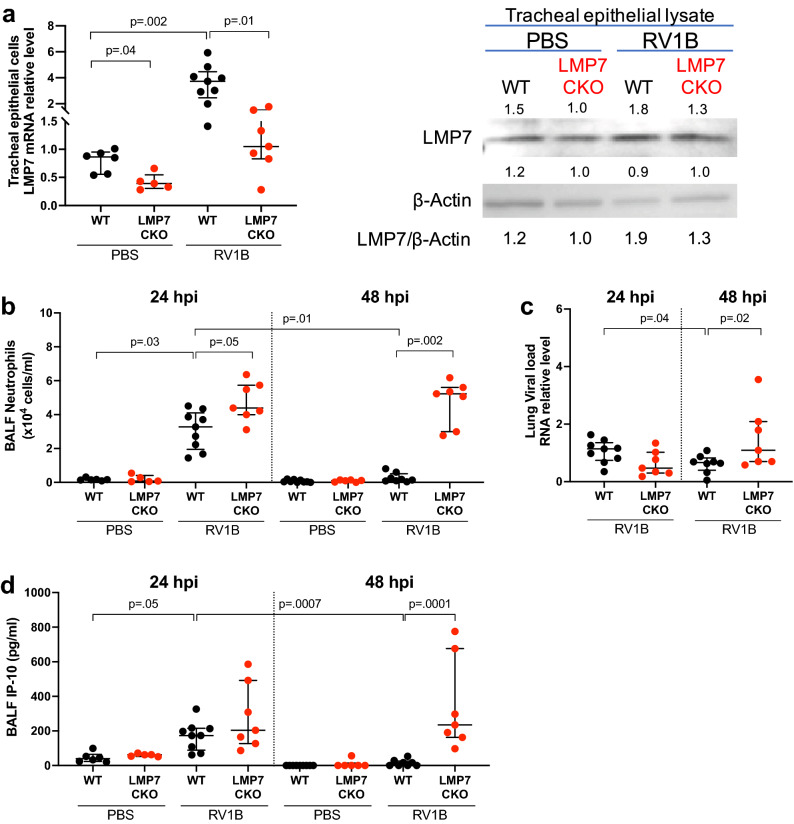
**LMP7 in mouse airway epithelium enhances the resolution of airway inflammation and viral infection**. Mice were intranasally inoculated with PBS or RV1B at 5 × 10^6^ PFU/mouse and sacrificed at 24h or 48h post infection. (**A**) Relative level of LMP7 mRNA from tracheal epithelial cells isolated from individual mice, and western blot image of LMP7 protein in tracheal epithelial lysates pooled from mice in the same groups. (**B**) Total number of neutrophils in BALF. (**C**) RV load was measured in the lung by RT-qPCR and normalized to 18S rRNA gene. (**D**) IP-10 levels in BALF. Data were analyzed using Kruskal–Wallis test. n = 5–9 mice per group.

To establish the in vivo function of airway epithelial LMP7 in lung viral infection, we generated tamoxifen-inducible LMP7 CKO mice that have the *Sox2* open reading frame. *Sox2* is a transcription factor that is important for epithelial cell differentiation and proliferation. In mouse lungs, *Sox2* is exclusively expressed in the conducting airway epithelial cells^23^. Feeding with tamoxifen chow induces Cre expression with subsequent deletion of LMP7 in the airway epithelial cells. We confirmed 55% decrease in LMP7 mRNA in isolated tracheal epithelial cells from individual LMP7 CKO mouse (median = 0.39, interquartile range (IQR) 0.30–0.54) compared to wild-type (WT) mice (median = 0.87, IQR 0.55–0.95) (Fig. 1a). This was supported by the lower LMP7 protein expression in the pooled tracheal epithelial cell lysate from the LMP7 CKO (n = 6) vs. WT mice (n = 5). As expected, RV upregulated LMP7 mRNA and protein expression in the WT mice. Importantly, LMP7 CKO mice challenged with RV1B failed to significantly increase LMP7 mRNA and protein levels.

At 24h post RV infection, neutrophil levels in bronchoalveolar lavage fluid (BALF) were significantly higher in the LMP7 CKO mice compared to the WT mice. In WT mice, neutrophil levels were reduced at 48h vs. 24h post infection while LMP7 CKO mice maintained a high level of neutrophilic inflammation during 48h of infection (Fig. 1b). We did not observe any significant difference in macrophages when comparing LMP7 CKO and WT mice (Supplementary Fig. 1) while lymphocyte count was low in all groups. Lung viral load was in line with the neutrophil data wherein the viral load was significantly reduced from 24h to 48h post infection in WT mice, but increased in the LMP7 CKO mice (Fig. 1c). This result was also supported by the higher level of IP-10 (CXCL10, a chemokine induced by active viral infection) in BALF of RV1B-infected LMP7 CKO mice at 48h (Fig. 1d). Our data suggests that airway epithelial LMP7 is critical to the resolution of neutrophilic inflammation and viral infection.

### LMP7 deficiency in primary human airway epithelial cells increases pro-inflammatory responses and viral load

**Figure 2:**
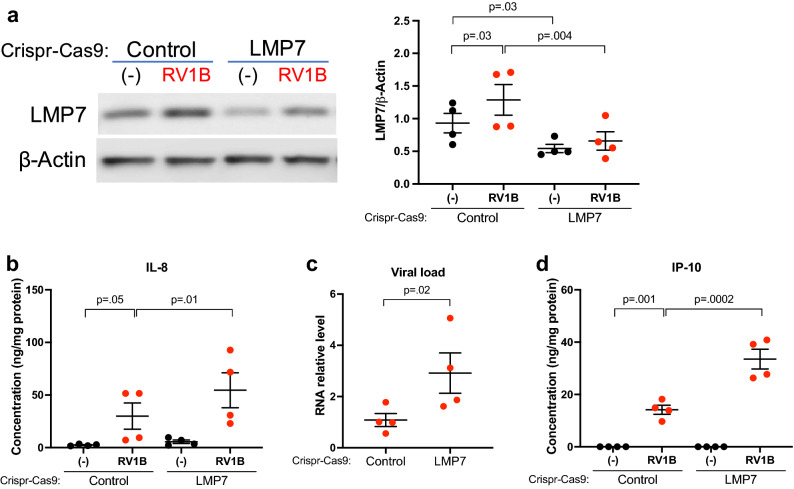
**LMP7 inhibits pro-inflammatory responses and reduces viral load in RV-infected human tracheobronchial epithelial (HTBE) cells**. HTBE cells (n = 4 independent experiments) were transduced with lentivirus containing the LMP7 sgRNA or scramble control (Control) plasmid construct and were differentiated at air–liquid interface (ALI) culture. Cells were infected with 10^5^ PFU/well RV1B or 0.1% BSA-PBS (−) at day 21 of ALI. (**A**) Representative western blot image and densitometry of LMP7 protein. (**B**) IL-8 levels measured in the basolateral supernatant by ELISA were normalized to total protein in the cell lysate. (**C**) RV load was quantified by RT-qPCR and normalized to GAPDH gene. (**D**) IP-10 levels measured in the basolateral supernatant by ELISA were normalized to total protein levels in the cell lysate. Data were analyzed using one-way ANOVA with Holm–Sidak’s test (**A**,**B**,**D**) and Student’s t test (**C**).

Having demonstrated the role of airway epithelial LMP7 in vivo, we sought to verify the function of LMP7 in primary human airway epithelial cells. LMP7 CRISPR (mean ± SEM = 0.54 ± 0.06) vs. Control CRISPR (mean ± SEM = 0.93 ± 0.15) HTBE cells demonstrated significantly lower level of LMP7 protein expression at the baseline, thus confirming deficiency in LMP7 expression of about 42% (Fig. 2a). Similar to what we found in vivo, RV1B significantly increased LMP7 protein expression in Control CRISPR cells but not in the LMP7 CRISPR cells. Importantly, we found significant increase in the neutrophilic chemoattractant IL-8 (Fig. 2b) as well as viral load (Fig. 2c) in LMP7-deficient cells infected with RV1B. The chemokine IP-10 (Fig. 2d) in the basolateral supernatant was also significantly increased in RV1B-infected LMP7 CRISPR cells. These findings further suggest that airway LMP7 exerts both anti-inflammatory and antiviral functions against RV infection.

### LMP7 upregulation by a low dose of PI:C reduces neutrophilic inflammation in RV-infected mouse airways

**Figure 3:**
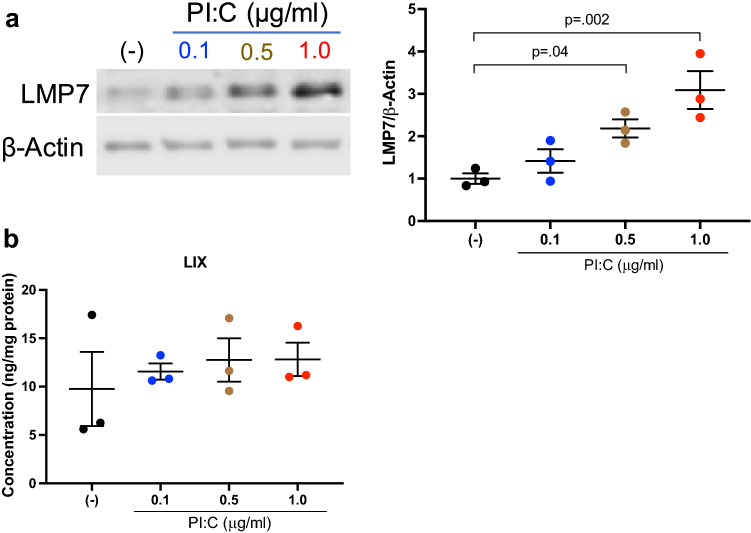
**PI:C (1 µg/ml) upregulates LMP7 expression with minimal pro-inflammatory effects in cultured mouse tracheal epithelial cells (mTECs)**. mTECs (n = 3 independent experiments) in submerged culture were stimulated with different doses of PI:C (0.1, 0.5, 1.0 μg/ml) or medium control (−) for 24h. (**A**) Representative western blot image and densitometry of LMP7 protein. (**B**) LIX levels in the supernatant were normalized to total protein in the cell lysate. Data were analyzed using one-way ANOVA with Holm–Sidak’s test.

We next investigated whether appropriate induction of LMP7 in the lung may be used as a potential therapy to reduce RV-mediated inflammation. Agonists of toll-like receptors (TLRs) such as PI:C, a viral mimic, are currently being explored as potential adjuvants for vaccines as well as for prophylaxis^24,25^. Kumaki et al.^25^ reported that a lower dose of PI:C compared to a higher dose is more effective in reducing the severity of SARS-CoV-1 infection. However, the role of PI:C in induction of IP such as LMP7 has not been tested. By performing a dose response of PI:C in WT mouse tracheal epithelial cells, we found that 1 μg/ml PI:C stimulation induced a threefold increase in LMP7 expression (Fig. 3a) without a pro-inflammatory effect as indicated by no induction of a neutrophil chemokine LIX (Fig. 3b).

**Figure 4:**
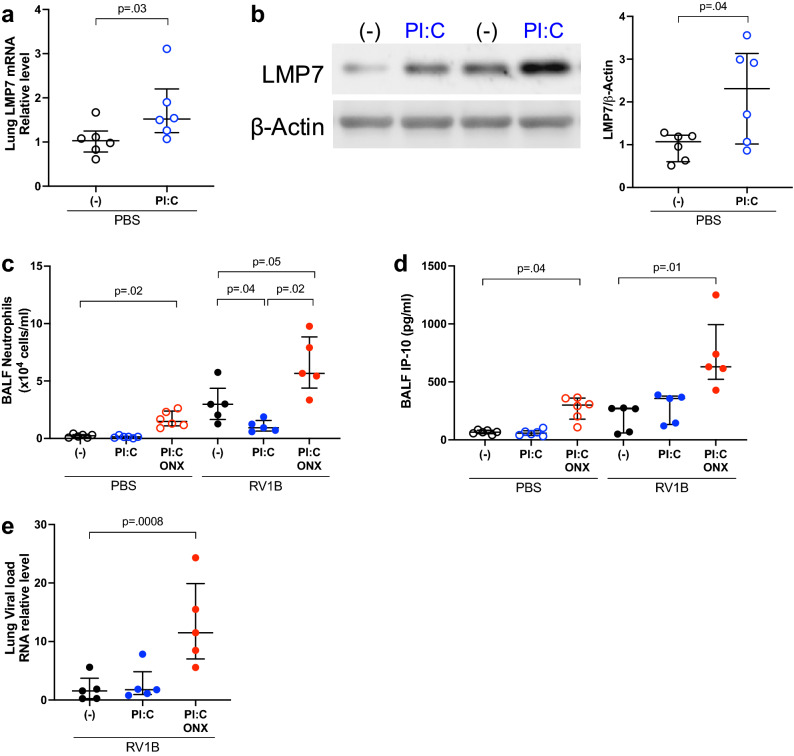
**A low dose of PI:C reduces neutrophilic inflammation in wild-type (WT) mice infected with RV1B**. Mice were pre-treated with 2 μg/mouse PI:C or PBS intranasally. At 20h post PI:C treatment, mice were challenged with ONX-0914 at 5 mM/mouse or 0.5% DMSO-PBS via oropharyngeal administration for 4h followed by intranasal inoculation of RV1B at 5 × 10^6^ PFU/mouse or 0.1% BSA-PBS (PBS). Mice were sacrificed at 24h post infection. (**A**) LMP7 mRNA relative levels from individual mouse lungs were normalized to 18S rRNA gene. (**B**) Representative western blot image and densitometry of LMP7 protein. (**C**) Total number of neutrophils in BALF. (**D**) IP-10 levels in BALF. (**E**) RV load was measured in the lung by RT-qPCR and normalized to 18S rRNA gene. Data were analyzed using Mann–Whitney test (**A**,**B**) and Kruskal–Wallis test with Dunn’s multiple comparison test (**C**–**E**). n = 5–6 mice per group.

To determine if a low dose of PI:C can induce LMP7 expression and reduce RV-mediated neutrophilic inflammation in vivo, we pre-treated WT mice intranasally with PI:C at 2 μg/mouse in the presence or absence of a selective LMP7 inhibitor ONX-0914. Adding ONX-0914 to inhibit LMP7 activity allowed us to test if the effect of PI:C is dependent on IP. We observed that PI:C alone upregulated LMP7 mRNA (Fig. 4a) and protein expression (Fig. 4b) in the mouse lungs without increasing neutrophils (Fig. 4c) or IP-10 (Fig. 4d). This supports that a low dose of PI:C alone was effective in upregulating LMP7 without causing inflammation. We also did not observe any significant difference in macrophage counts in these mice (Supplementary Fig. 1). Following RV1B infection, the total number of neutrophils in the BALF was lower in mice treated with both PI:C and RV1B than those with RV1B infection alone. However, PI:C did not alter the BALF IP-10 as well as the viral load (Fig. 4e) in the RV1B-infected mice. Importantly, ONX-0914 administration in infected mice resulted in higher levels of neutrophils, IP-10 and viral load compared to mice infected with RV1B alone, supporting the anti-inflammatory and antiviral function of LMP7.

**Figure 5:**
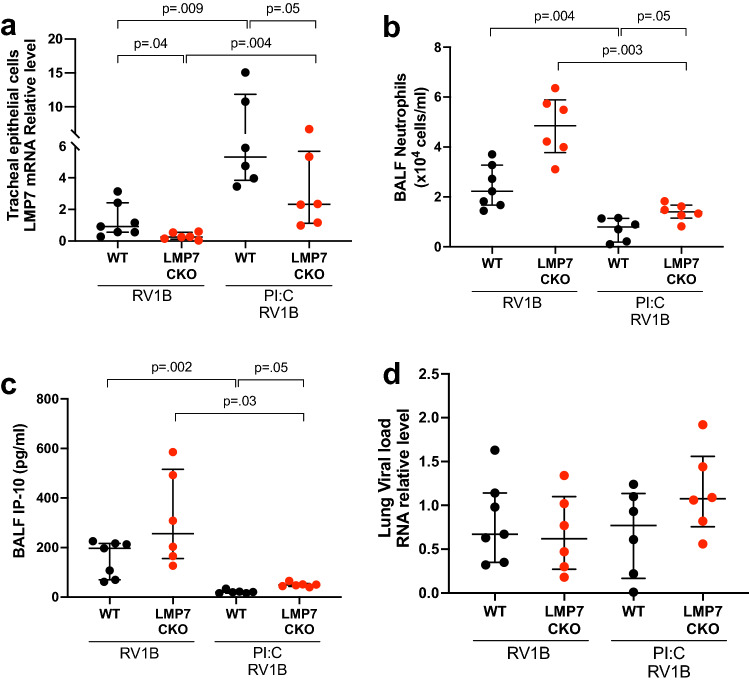
**A low dose of PI:C upregulates airway epithelial LMP7 and reduces neutrophilic inflammation in RV-infected LMP7 conditional knockout mice**. Mice were fed with tamoxifen chow for 7 days, and then regular chow for another 7 days. Mice were treated with 2 μg/mouse PI:C or PBS via intranasal inoculation for 24h followed by RV1B at 5 × 10^6^ PFU/mouse or 0.1% BSA-PBS (PBS). Mice were sacrificed at 24h post infection. (**A**) LMP7 mRNA relative levels in isolated tracheal epithelial from individual mice were normalized to 18S rRNA gene. (**B**) Total number of neutrophils in BALF. (**C**) IP-10 levels in BALF. (**D**) RV load was measured in the lung by RT-PCR and normalized to 18S rRNA gene. Data were analyzed using Kruskal–Wallis test. n = 5–7 mice per group.

We then sought to define the in vivo role of airway epithelial LMP7 induction by PI:C in RV1B infection using our airway epithelial LMP7 CKO mouse model. We found that PI:C enhanced the expression of LMP7 mRNA in isolated tracheal epithelial cells of RV1B-infected WT mice compared to WT mice infected with RV1B alone (Fig. 5a). Importantly, LMP7 CKO mice (median = 2.32, IQR 1.12–5.67) vs. WT mice (median = 5.32, IQR 3.84–11.84) challenged with both PI:C and RV1B showed about 60% less induction of LMP7 mRNA in airway epithelial cells. Less induction of LMP7 in the PI:C and RV1B-treated LMP7 CKO vs. WT mice was in line with increased number of BALF neutrophils (Fig. 5b) and IP-10 (Fig. 5c). Notably, LMP7 CKO mice that received PI:C following RV1B infection benefited from PI:C treatment by reducing the number of neutrophils and IP-10 in BALF compared to LMP7 CKO mice infected with RV1B without PI:C. Similar to what we found in the WT model, low dose PI:C did not alter the viral load (Fig. 5d) in the LMP7 CKO mice. Collectively, our data suggests that a low dose of PI:C primarily reduces neutrophilic inflammation with minimal effect on the viral clearance from the lung. Our findings also suggest that the therapeutic effect of PI:C is in part dependent on airway epithelial LMP7.

### LMP7 attenuates inflammation in RV1B-infected human airway epithelial cells by upregulating the negative immune regulator A20/TNFAIP3

To determine the molecular mechanism by which LMP7 may attenuate neutrophilic inflammation, we investigated the role of a negative immune regulator A20/TNFAIP3. A20 has been shown to be involved in lung epithelial defense against respiratory virus infection such as influenza A virus^26^. It is an anti-inflammatory protein that inhibits inflammation by suppressing NF-κB activation, a regulator of inflammation^27^. How A20 is regulated by LMP7 in the airway epithelium during RV infection has never been explored.

**Figure 6:**
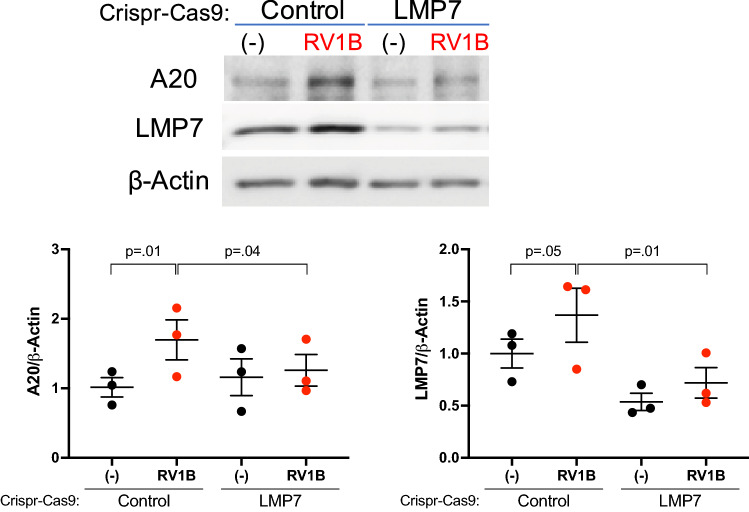
**LMP7 promotes A20 expression following RV infection**. HTBE cells (n = 3 independent experiments) were transduced with lentivirus containing the LMP7 sgRNA or scrambled control (Control) plasmid construct and were differentiated at air–liquid interface (ALI) culture. Cells were infected with 10^5^ PFU/well RV1B or 0.1% BSA-PBS (−) at day 21 of ALI. Representative western blot image and densitometry of A20 protein (90 kDa). Data were analyzed using one-way ANOVA with Holm–Sidak’s test.

**Figure 7:**
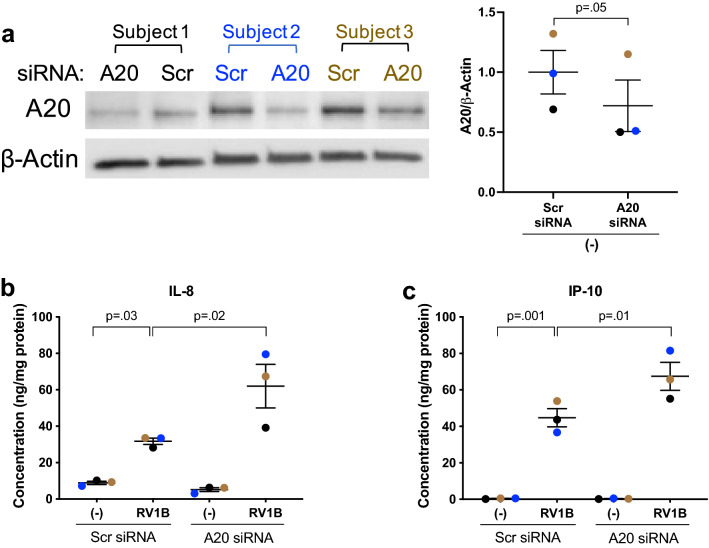
**A20 inhibits RV-mediated inflammation in human tracheobronchial epithelial (HTBE) cells**. HTBE cells (n = 3 donors) in submerged culture transfected with A20 siRNA or Scrambled control (Scr) siRNA were infected with RV1B at 5 × 10^4^ PFU/well or with 0.1% BSA-PBS (−) for 48h. (**A**) Representative western blot image and densitometry of A20 protein (90 kDa). (**B**) IL-8 and (**C**) IP-10 levels in the supernatant were normalized to total protein in the cell lysate. Horizontal bar represents mean. Data were analyzed using Student’s t test (**A**) and one-way ANOVA with Holm–Sidak’s test (**B**,**C**).

Using our airway epithelial CRISPR cells cultured at air–liquid interface, we observed the induction of A20 by RV1B infection in Control CRISPR cells but not in LMP7 CRISPR cells (Fig. 6). To further demonstrate the importance of A20 in response to RV infection, we carried out a gene knockdown experiment of A20 using siRNA in submerged culture of airway epithelial cells. As expected, A20 siRNA (mean ± SEM = 0.72 ± 0.21) vs Scrambled control (SCR) siRNA (mean ± SEM = 1.00 ± 0.18) reduced A20 protein expression at the baseline by about 30% (Fig. 7a). In A20 siRNA-transfected cells, RV1B infection resulted in a further increase in IL-8 (Fig. 7b) and IP-10 (Fig. 7c) compared to SCR siRNA-transfected cells. These data suggest a role of LMP7 in affecting A20 function or its stability during viral infection.

### LMP7 interacts with A20/TNFAIP3 in human primary airway epithelial cells

**Figure 8:**
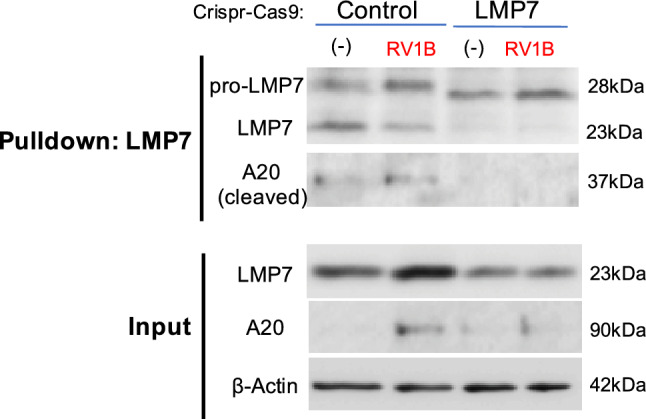
**Immunoproteasome subunit LMP7 interacts with A20 in Control CRISPR cells**. HTBE cells transduced with lentivirus containing the LMP7 sgRNA or scramble control (Control) plasmid construct were infected with 5 × 10^3^ PFU/well RV1B or 0.1% BSA-PBS (−) in submerged culture. Immunoblot of LMP7, A20 and β-Actin was done after performing an immunoprecipitation with LMP7.

To confirm if protein–protein interaction of LMP7 and A20 exists, we performed an LMP7 immunoprecipitation assay using LMP7-sufficient and LMP7-deficient primary HTBE cells generated by CRISPR-Cas9 system. In LMP7-sufficient cells, but not in LMP7-deficient cells, A20 was pulled down in the absence or presence of RV infection (Fig. 8). Specifically, we observed cleaved A20 (37 kDa) but not the full-length A20 (90 kDa) protein expression in the RV1B-infected LMP7-sufficient cells. Our data suggests the possible interaction of LMP7 and A20 in airway epithelial cells.

## Discussion

**Figure 9:**
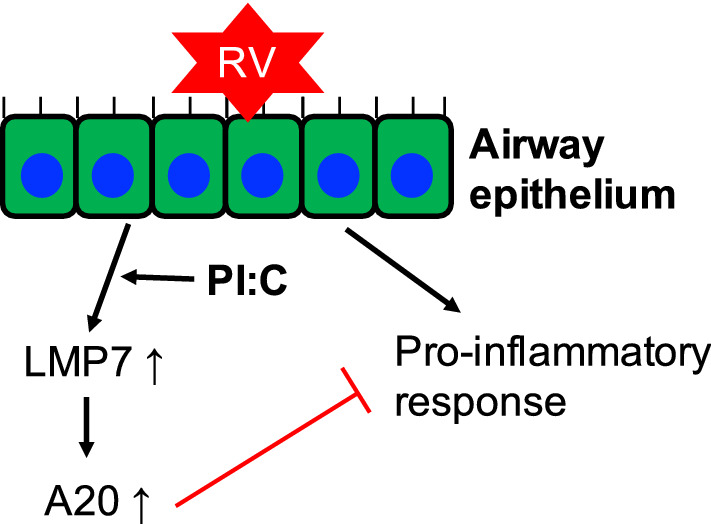
**Proposed mechanism of airway epithelial LMP7 during rhinovirus (RV) infection**. LMP7 induction by RV infection inhibits pro-inflammatory response through A20 upregulation. Also, administration of therapeutic low dose of polyinosinic:polycytidylic acid (PI:C) induces LMP7 to reduce RV-mediated inflammation.

The present study has revealed a novel role of airway epithelial LMP7 in RV-mediated airway inflammation (Fig. 9). Specifically, we demonstrated that LMP7 exerts anti-inflammatory and antiviral functions that contribute to the resolution of inflammation and decreased viral load during RV infection. We also uncovered that induction of LMP7 by administrating a low dose of PI:C reduces RV-mediated neutrophilic inflammation in vivo. Lastly, we demonstrated that LMP7 promotes the expression of a negative immune regulator A20 and interacts with A20, which may contribute to the anti-inflammatory function of LMP7.

The airway epithelium acts as the first line of defense against invading pathogens and environmental insults because of its broad spectrum of functions related to inflammation, immunity and host defense^28^. The innate immune mechanisms of the airway epithelium such as barrier function to evade pathogens and secretion of antimicrobial proteins for host defense are being explored due to its contribution in the pathogenesis of lung diseases (e.g. asthma and COPD)^28,29^. In asthma, RV-induced exacerbations are a major cause of disease morbidity and mortality, and increasing healthcare costs^30^. Airway epithelial cells, the primary target of respiratory viruses such as RV, can mount both inflammatory and antiviral innate immune responses aimed to efficiently clear the virus^31^. In our previous study, we provided the first evidence that RV infection in human primary airway epithelial cell culture and mouse models induces the expression of IP and this induction enhances the host antiviral mechanism^9^.

In this study, we, for the very first time, have delved deeper into the anti-inflammatory and antiviral function of LMP7 by using a newly generated mouse model of inducible LMP7 deficiency specifically in the airway epithelium. In addition, we also used a complementary in vitro model using LMP7-deficient primary human airway epithelial cells generated by CRISPR-Cas9. Using these two robust approaches, we provided strong data supporting that LMP7 specifically in the airway epithelium enhances the resolution of inflammation against RV infection by exerting anti-inflammatory and antiviral functions. Furthermore, we demonstrated that inducing LMP7 expression using a low dose of PI:C reduced RV-mediated neutrophilic inflammation in vivo. PI:C has been shown to induce interferon (IFN) signaling^32,33^. In our previous study^9^, we identified that IFN-λ is an upstream regulator of IP induction in primary human airway epithelial cells. This mechanism may in part support how PI:C-induced LMP7 expression contributes to the anti-inflammatory effect of IP; although a lower dose of PI:C may have limited effect on robust IFN induction in our model. Nevertheless, this highlights the beneficial effects of inducing LMP7 in the airway epithelium against viral infection.

It has been demonstrated that deficiency in IP leads to increased inflammation. Briefly, Opitz et al. showed that IP-deficient cells had impaired NF-κB activation leading to excessive inflammation and tissue injury in a coxsackievirus-induced myocarditis model^8^. This is also in concordance with increased pro-inflammatory cytokines in IP-deficient mice infected with *Toxoplasma gondii*^34^. Thus, how LMP7 subunit exerts its anti-inflammatory function during RV infection has never been investigated. An exciting finding from our study is the protein–protein interaction of LMP7 with a negative immune regulator A20/TNFAIP3. A20 is an endogenous negative regulator of NF-κB signaling. It is a ubiquitin-editing enzyme that can function to either remove K63-linked polyubiquitin chains or add K48-linked polyubiquitin chains on different substrates to restrict NF-κB signaling (i.e. RIPK1, TRAF6, NEMO)^35^. The 90 kDa A20 protein can be cleaved into two fragments: A20p50 (50 kDa) and A20p37 (37 kDa)^36^. In the present study, we observed more cleaved A20 or A20p37 in RV1B-infected LMP7-sufficient cells in our LMP7 immunoprecipitation assay. Coornaert et al.^36^ demonstrated that A20p37 had inhibitory function against NF-κB activation compared to A20p50. This suggests that the recruitment of A20p37 by LMP7 may inhibit NF-κB activation contributing to the anti-inflammatory function of LMP7 during RV infection. Similar to what others have shown^37,38^, we also found that silencing A20 increased IL-8 and IP10 levels in airway epithelial cells infected with RV1B. Importantly, we found that deficiency of LMP7 resulted in lower A20 expression after RV infection, which could explain why RV-mediated inflammation (i.e. IL-8 and neutrophils) is higher in the airway epithelial LMP7-deficient in vivo and in vitro models. Therefore, regulation of A20 expression and function by LMP7 may serve as a new mechanism in the resolution of RV-induced inflammation. However, the exact mechanism by which LMP7 upregulates A20 still warrants future studies. For example, we do not know whether other IP subunits will also interact with A20. It will be interesting to know if A20 will be able to interact with any of the IP subunits when a proteasome inhibitor is used. Nevertheless, we hypothesized that LMP7 upregulates A20 in airway epithelial cells as a protective mechanism against RV-induced inflammation. IP has been reported to have function in protein homeostasis by preventing accumulation of damaged proteins^39^. During RV infection, viral replication enhanced the production of misfolded and damaged proteins leading to IP induction for efficient degradation. Upregulation of A20 in part due to ER stress was also shown by Wang et al.^40^. It may be possible that LMP7 induction during ER stress upregulates A20, which needs to be tested in future studies.

There are several interesting findings in our study. First, we observed no difference in the lung viral load after low dose PI:C treatment in our WT and CKO mouse models. Although low dose PI:C treatment reduced lung neutrophilic inflammation during RV infection, it did not significantly reduce the viral load. Previous studies demonstrated that asthmatics with RV-associated asthma exacerbations did not show increased viral load as compared to healthy control subjects with viral infection^41,42^, suggesting that viral load is not a major factor contributing to the disease severity. Instead, excessive pro-inflammatory response such as increased neutrophils in sputum samples of asthmatics^41^ may contribute to the severity of disease. Second, we focused on the contribution of airway epithelial cells to the beneficial effect of a low dose of PI:C, but the contribution of other cell types including lung macrophages may need to be explored in the future. Third, we observed the interaction between A20 and LMP7 in our cell culture but it remains unclear whether A20 interacts with LMP7 alone or with the IP complex or other IP subunits. Additionally, how this interaction affects pro-inflammatory signaling such as NF-κB activation deserves further investigation.

Limitations in the current study are that we did not study the potential effect of PI:C on other immune functions such as induction of interferons^43,44^ as well as the induction of LMP7 activity (vs. expression) in the airway epithelial cells or other types of cells (e.g. macrophages or T cells) after RV infection. Understanding the multiple mechanisms of low dose of PI:C in the treatment of rhinovirus infection may maximize the beneficial effects while avoiding the potential side effects.

In conclusion, we showed that airway epithelial LMP7 has anti-inflammatory and antiviral functions that are important for the resolution of inflammation during RV infection. Understanding the contribution of LMP7 in the development of airway diseases, such as asthma, may provide a novel avenue for therapeutic intervention in improving the health of patients with viral-induced asthma exacerbations.

## Methods

### RV1B propagation

RV1B (American Type Culture Collection, Manassas, VA) were propagated in H1-Hela cells (CRL-1958, ATCC), purified and titrated to plaque-forming unit (PFU) as described previously^9^.

### Generation of inducible airway epithelial LMP7 conditional KO mice and infection with RV1B

The model design and generation of LMP7 fl/fl mice were performed by genOway (Lyon, France) as part of a project of Dr. Frank Heppner and Elke Krüger at the Universitätsmedizin Greifswald, Germany. In brief, a loxP-flanked LMP7 exon 3 containing an FRT-flanked neomycin cassette was introduced into the endogenous LMP7 locus by homologous recombination. Targeted embryonic stem cells were used to generate LMP7fl-neo mice. Flp-mediated recombination resulted in the deletion of the neomycin selection cassette (LMP7fl mice). With the LMP7fl mice, the Cre-loxP recombination approach was used to generate airway epithelial LMP7-specific conditional knockout (CKO) mice expressing *Cre* recombinase under the *Sox2* promoter. Briefly, Sox2-CreER knockin mice was purchased from Jackson Laboratory. These mice were crossed and inbred for at least four generations to finally produce Sox2-Cre + LMP7 mice, which were used as LMP7 CKO mice, and Sox2-Cre- LMP7 mice, which were used as WT or control mice. In order to induce the *Cre *recombinase gene or the specific deletion of LMP7 in the epithelial cells, mice were fed with tamoxifen chow (Envigo) for 7 days. Afterwards, mice were fed with regular chow (Envigo) for another 7 days before infecting with RV1B at 5 × 10^6^ PFU/mouse or 0.1% BSA-PBS (control) via intranasal inoculation for 24h or 48h. Mice were anesthetized by intraperitoneal injection of ketamine (80 mg/kg) and xylazine (10 mg/kg). Bronchoalveolar lavage fluid (BALF) was collected by lavaging the mouse lungs with 1 ml sterile saline. Cell differentials were counted from cytospin slides of BALF cells while cell-free BALF was used to assess pro-inflammatory mediators by ELISA. The left lung lobe was placed in RNALater (Thermo Fisher Scientific) for RNA extraction using TRIzol reagent (Thermo Fisher Scientific). Lastly, trachea from each mouse was excised and digested in DMEM with 0.1% protease (Sigma-Aldrich) and 50 µg/mL amphotericin B (Sigma-Aldrich) at 37 °C for 1h to isolate mouse tracheal epithelial cells. Cells were washed and collected to characterize LMP7 deficiency by qRT-PCR from individual mouse trachea or by western blot from the pooled tracheas of all mice.

All animal studies were approved by the Institutional Animal Care and Use Committee (IACUC) at National Jewish Health (protocol# AS2972-03-23). All experiments were conducted according to the ARRIVE guidelines as well as the guidelines and regulations of the IACUC at NJH. Both male and female mice were used in all mouse experiments.

### Culture of LMP7-deficient primary human tracheobronchial epithelial (HTBE) cells at air- liquid interface (ALI)

Detailed method on isolation and culturing of primary HTBE cells from normal healthy donors were described in our previous publications^9,45^. LMP7-deficient cells were generated using the CRISPR-Cas9 method as we previously described^46^. Scrambled sgRNA was used for the control CRISPR while single guide RNA (5′CCAGAGCTCGCTTTACCCCG 3′) targeting exon1 of human LMP7 was used in HTBE cells.

Successful transduction and expansion of control CRISPR and LMP7 CRISPR in HTBE cells were seeded in 12-well-transwell plates (Transwell 2460) with PneumaCult-ALI medium (StemCell). After 7 days of submerged culture, cells were shifted to air liquid interface (ALI) for the next 21 days to induce mucociliary differentiation. Differentiated cells were infected in the apical surface with RV1B at 10^5^ PFU/well or 0.1% BSA-PBS (control) for 2h. Viruses were removed and the ALI culture was further incubated to 48h at 37 °C. We collected the cells and basolateral supernatants at 48h post infection based on the cell culture results from our previous publication^9^. Cells were lysed with RLT buffer for RNA extraction and with RIPA buffer for Western blot analysis. Basolateral supernatant was used for ELISA.

### Mouse tracheal epithelial cells isolation and culture

Tracheas were excised from WT C57BL/6 mice as previously described^9^. Cells were grown in 24-well plate and stimulated with different doses (0.1, 0.5 or 1.0 μg/ml) of PI:C for 24h. Cells and supernatant were harvested. Cells were lysed with RIPA buffer for Western blot analysis while supernatant was used for ELISA.

### Mouse models of a low dose of PI:C treatment with RV1B infection

We used 2 μg/mouse of PI:C in our mouse model based on our cell culture result using mouse tracheal epithelial cells wherein we showed that 1 μg/ml PI:C can induce LMP7 protein expression with minimal pro-inflammatory response. We speculated that intranasally administering 2 μg/mouse of PI:C in a 50 µl volume would deposit about 1 μg PI:C (~ 50%) in the airways as shown by previous reports of Southam et al.^47^ and Eyles et al.^48^.

We used WT C57BL/6 mice purchased from The Jackson Laboratory to determine if PI:C can induce airway LMP7 reduce neutrophilic inflammation and viral load. Mice were intranasally inoculated with 2 μg/mouse PI:C or PBS (control) for 2h. At 20h post PI:C treatment, mice were pre-challenged with ONX-0914 (APExBIO) at 5 mM/mouse or 0.5% DMSO-PBS (control) via oropharyngeal administration for four hours. This was followed with intranasal inoculation of RV1B at 5 × 10^6^ PFU/mouse or 0.1% BSA-PBS (control).

Additionally, we also investigated the effect of low dose PI:C in our CKO mouse model using Sox2-Cre + LMP7 mice and Sox2-Cre− LMP7 mice to demonstrate the dependency of the low dose PI:C on airway epithelial-specific LMP7. Mice were intranasally inoculated with 2 μg/mouse PI:C or PBS (control) for 24h followed by intranasal inoculation of RV1B at 5 × 10^6^ PFU/mouse or 0.1% BSA-PBS (control).

All mice were sacrificed 24h post RV infection. Cell differentials were counted from cytospins of BAL cells. Cell-free BALF was used to assess pro-inflammatory mediators by ELISA. Lastly, the left lung lobe was place in RNALater (Thermo Fisher Scientific) for RNA extraction using TRIzol reagent (Thermo Fisher Scientific).

### Transfection of A20 small interfering RNA (siRNA) in human airway epithelial cells

HTBE cells (n = 3 donors) in 24-well plates (5 × 10^4^ cells/well) under submerged culture were transfected with 200 nM A20 siRNA (ID: 12159; Thermo Fisher Scientific) or with Scrambled (Scr) control siRNA using Lipofectamine RNAiMax transfection reagent (Thermo Fisher Scientific) according to manufacturer’s protocol. After 48h of transfection, cells were infected with RV1B at 5 × 10^4^ PFU/well (MOI = 1) or with 0.1% BSA-PBS (control) at 37 °C, 5% CO2 for 2h. Cells were washed with PBS to remove the virus. Cells and supernatant were harvested at 48h post infection. Cells were lysed with RIPA buffer for Western blot analysis while supernatant was used for ELISA.

### Quantitative real-time reverse-transcription PCR

RNA extracted using the GenCatch Total RNA Extraction System (Epoch Life Sciences) was reversely transcribed to cDNA. mRNA levels of LMP7 were measured using Taqman gene expression assay (Thermo Fisher Scientific). RV sequence (FW: 5′ CCTCCGGCCCCTGAAT 3′; RV: 5′ GGTCCCATCCCGCAATT 3′; probe: 5′ CTAACCTTAAAC CTGCAGCCA 3′) was purchased from Integrated DNA Technologies. To determine mRNA relative expression levels, the comparative cycle of threshold (ΔΔCT) method was performed with the housekeeping gene 18S rRNA as an internal control to normalize the expression of a target gene.

### Western blot

Cell lysates were prepared and analyzed as previously described^46^. Blots were probed with antibody against LMP7 (1:1000, Proteintech), A20 (1:1000, Novus) or β-actin (1:500, Santa Cruz Biotechnology) followed by horseradish peroxidase-conjugated secondary IgG (1:3000; EMD Millipore, Burlington, Massachusetts, USA). Densitometry was performed using the National Institutes of Health’s ImageJ software.

### Immunoprecipitation

Cells were harvested with RIPA buffer and sonicated. After centrifugation, cell lysate was pre-cleared with protein A/G magnetic beads (MedChemExpress) for 20 min at room temperature. Rabbit anti-LMP7 antibody (Peprotech) with the pre-cleared lysate was incubated for 24h at 4 °C. The magnetic beads were then added and further incubated for 24h at 4 °C. Immunoprecipitated proteins were separated on 12% SDS-PAGE for Western blot analysis of rabbit anti-LMP7 (Peprotech), mouse anti-A20 (Novus) and mouse anti-actin (Sta. Cruz).

### ELISA

Human IL-8 and mouse CXCL5/LIX were measured using Duoset ELISA kit (R&D Systems) while human and mouse CXCL10/IP10 were measured using ABTS ELISA Development kit (Peprotech) according to manufacturer’s specifications. ELISA results from cell culture supernatant were normalized to protein content of cell lysate.

### Statistical analyses

GraphPad Prism version 9.0 software was used for all statistical analyses. Shapiro Wilk test was used as a normality test to determine distribution of the data. For parametric tests, a Student’s t-test was performed for two-group comparisons while one-way analysis of variance (ANOVA) with Holm–Sidak’s post hoc test was done for multiple comparisons. Data were presented as mean ± standard error of the mean (SEM). All data from mouse studies, due to the nonparametric nature, were presented as median with interquartile range (IQR). Comparisons were done using Mann–Whitney test for two group comparisons while Kruskal–Wallis test with Dunn’s test for multiple comparisons. A probability value < 0.05 was considered statistically significant.

## Supplementary Information

Supplementary Information 1.

Supplementary Information 2.

## Data Availability

All data generated or analyzed during this study are included in this published article.
