# Supplementary figures and images for: Airway epithelial immunoproteasome subunit LMP7 protects against rhinovirus infection
Source: Sci Rep. 2022 Aug 25;12:14507. doi: 10.1038/s41598-022-18807-3 (PMC9403975; doi:10.1038/s41598-022-18807-3)

**Supplementary Figure 1.** Macrophage data from mouse bronchoalveolar lavage fluid (BALF)

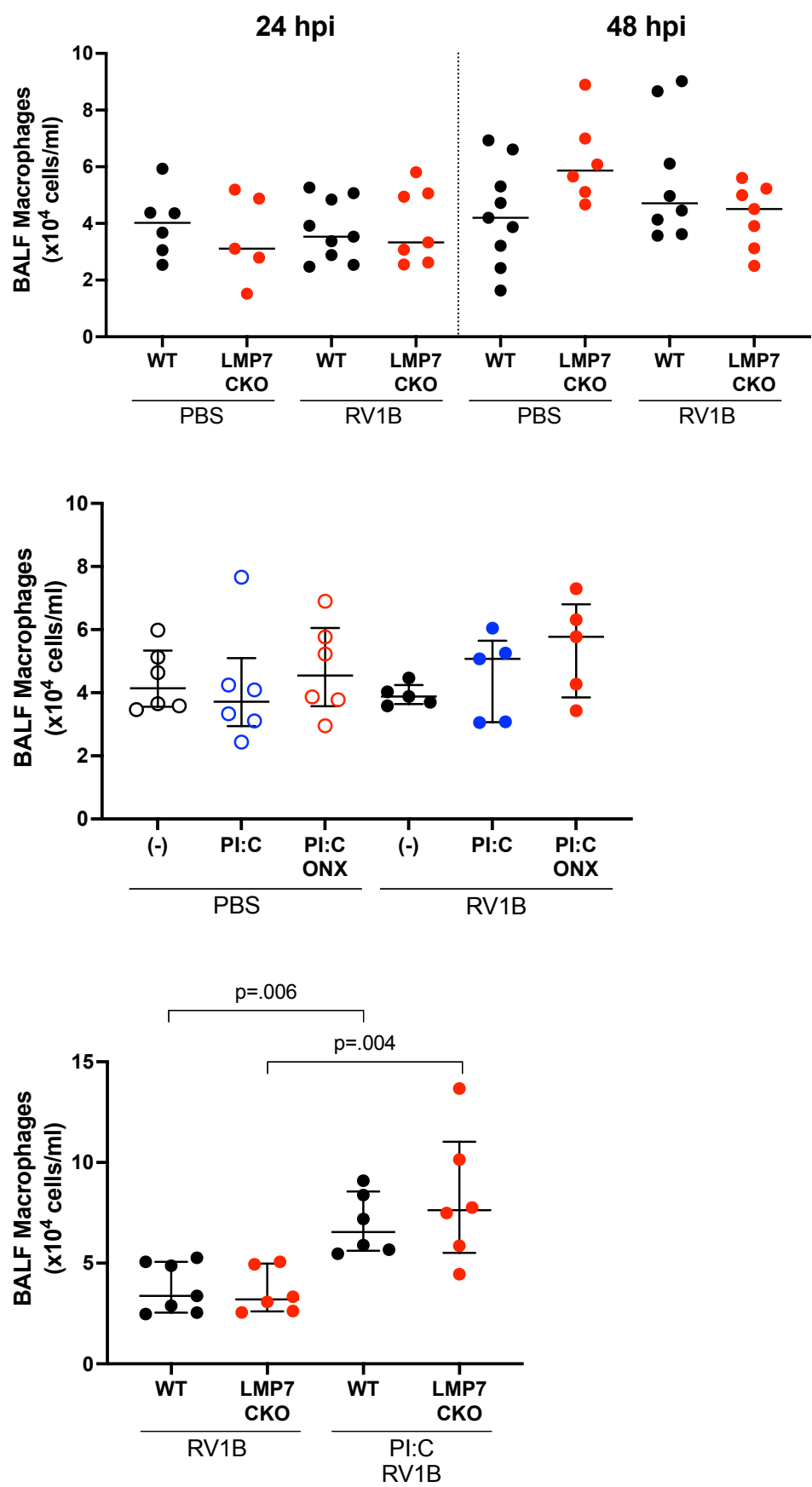

Supplement: Supplementary file 1 — Supplementary Information 1. [file 41598_2022_18807_MOESM1_ESM.pdf]
