# Supplementary material for: Airway epithelial immunoproteasome subunit LMP7 protects against rhinovirus infection
Source: Sci Rep. 2022 Aug 25;12:14507. doi: 10.1038/s41598-022-18807-3 (PMC9403975; doi:10.1038/s41598-022-18807-3)
Supplement: Supplementary file 2 — Supplementary Information 2. [file 41598_2022_18807_MOESM2_ESM.pdf]

Figure 1a. LMP7 and β-actin western blot

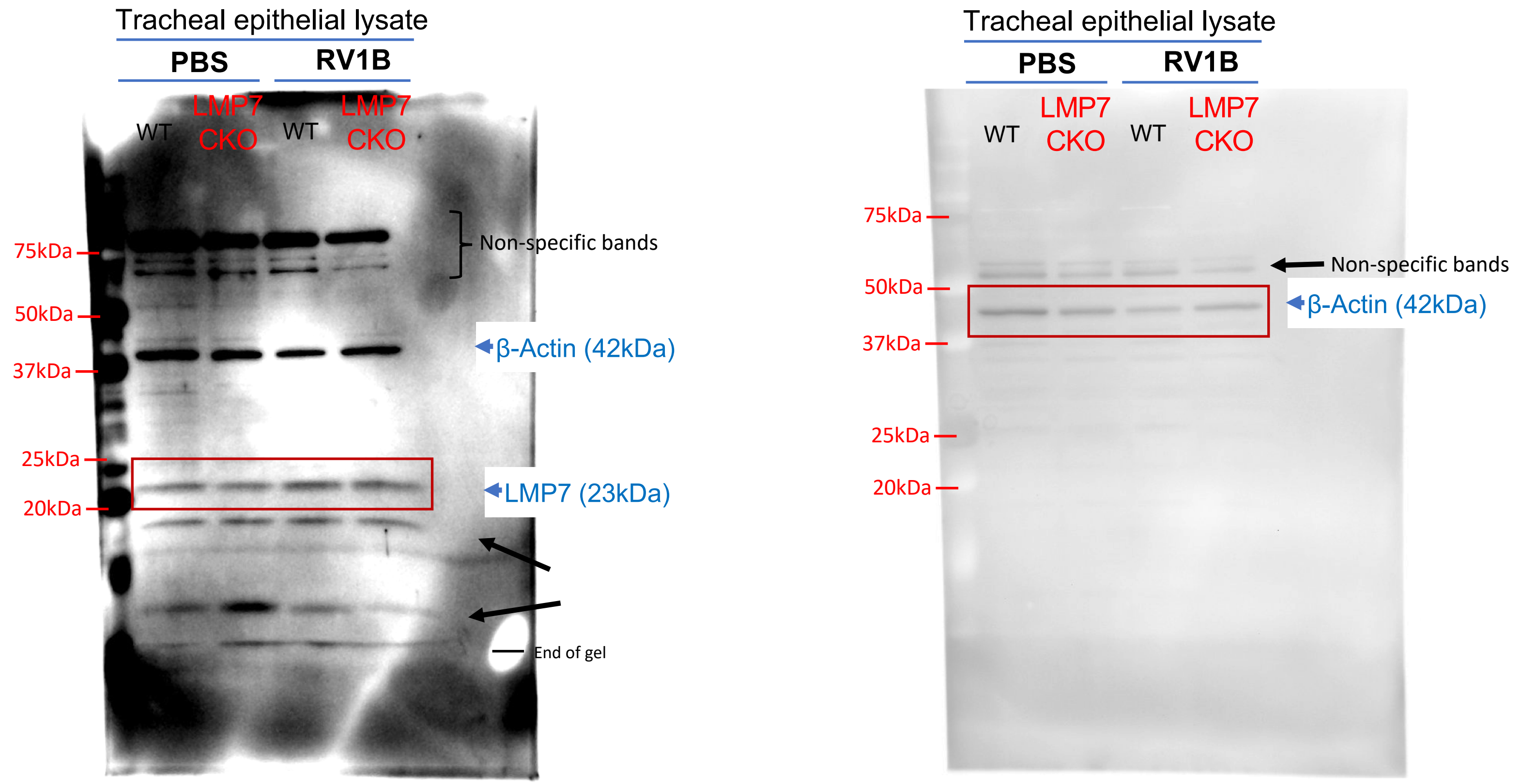

Membrane was probed first with β-actin followed by LMP7.
Bands were visualized using chemiluminescence (Amersham ECL Prime Western Blotting Detection Reagent) on a FOTO/Analyst® LuminaryFX Workstation.
Bands were quantified using ImageJ.
Note: As β-Actin signal is stronger than LMP7, we used the β-Actin image from the right panel (not saturated) for Figure 1a.

Figure 2a. LMP7 and β-actin western blot

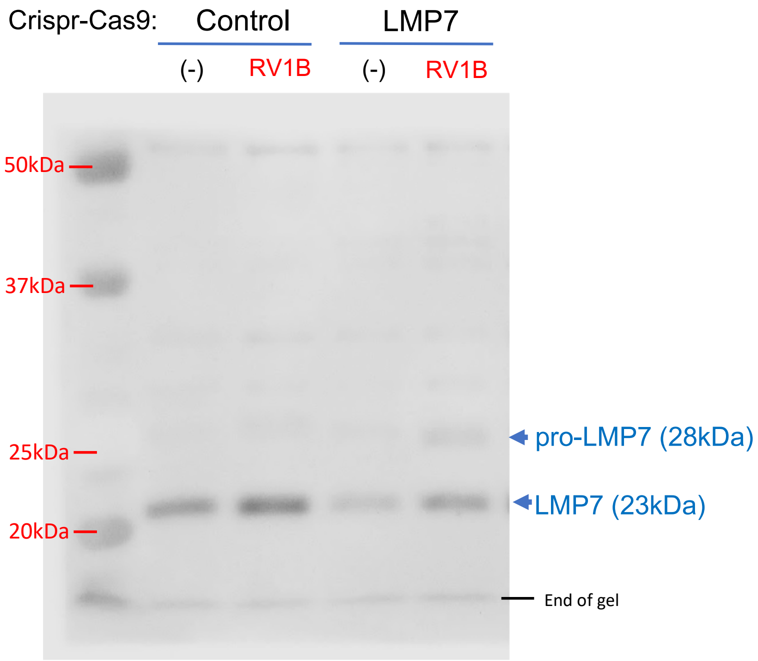

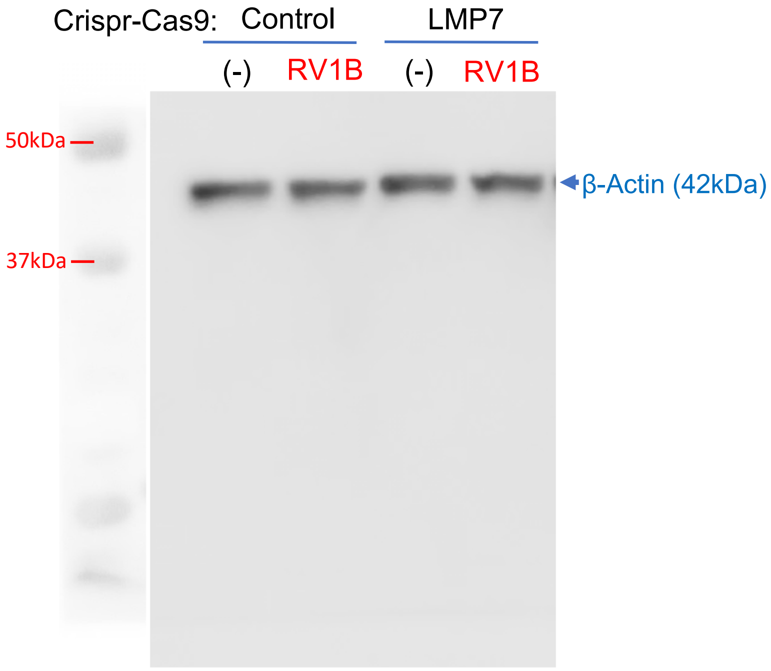

Membrane was cut between 50kDA (lower membrane) and 75kDa bands (upper membrane). The lower membrane was used for LMP7 and β-actin western blots.
Bands were visualized using chemiluminescence (Amersham ECL Prime Western Blotting Detection Reagent) on a FOTO/Analyst® LuminaryFX Workstation.
Bands were quantified using ImageJ.

Figure 3a. LMP7 and β-actin western blot

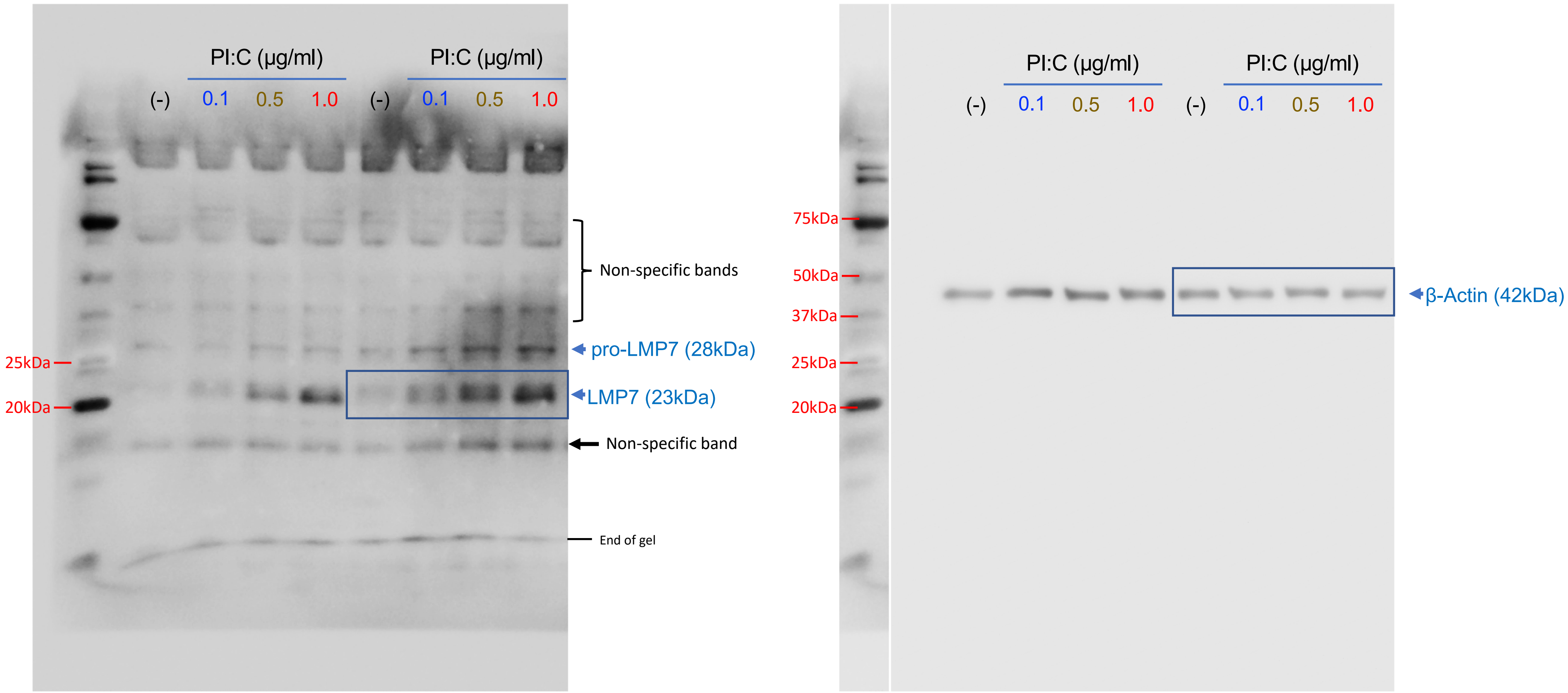

Bands were visualized using chemiluminescence (Amersham ECL Prime Western Blotting Detection Reagent) on a FOTO/Analyst® LuminaryFX Workstation.
Bands were quantified using ImageJ.

Figure 4b. LMP7 and β-actin western blot

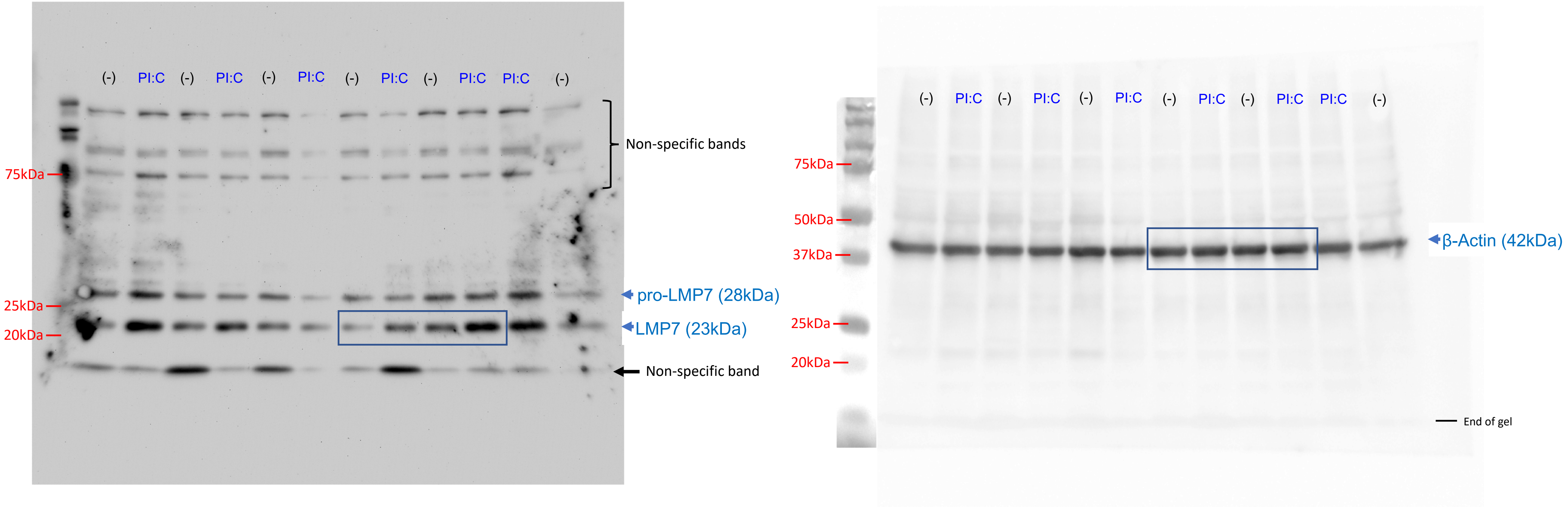

Bands were visualized using chemiluminescence (Amersham ECL Prime Western Blotting Detection Reagent) on a FOTO/Analyst® LuminaryFX Workstation.
Bands were quantified using ImageJ.

Figure 6. A20, LMP7 and β-actin western blot

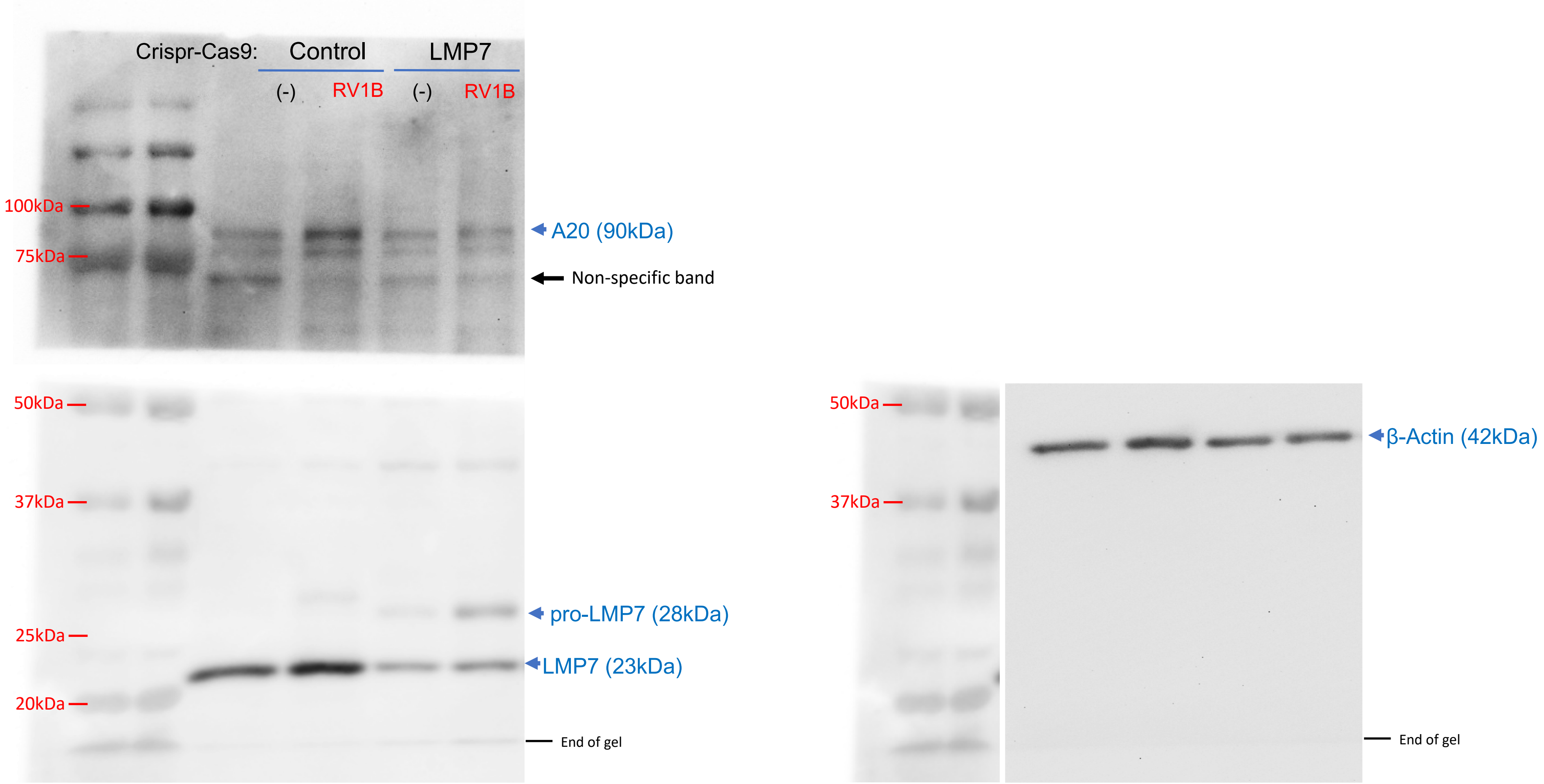

Membrane was cut between 50kDA (lower membrane) and 75kDa bands (upper membrane).
The upper membrane was used to probe for A20 while the lower membrane was used for LMP7 and β-actin western blots.
Bands were visualized using chemiluminescence (Amersham ECL Prime Western Blotting Detection Reagent) on a FOTO/Analyst® LuminaryFX Workstation.
Bands were quantified using ImageJ.

Figure 7a. A20 and β-actin western blot

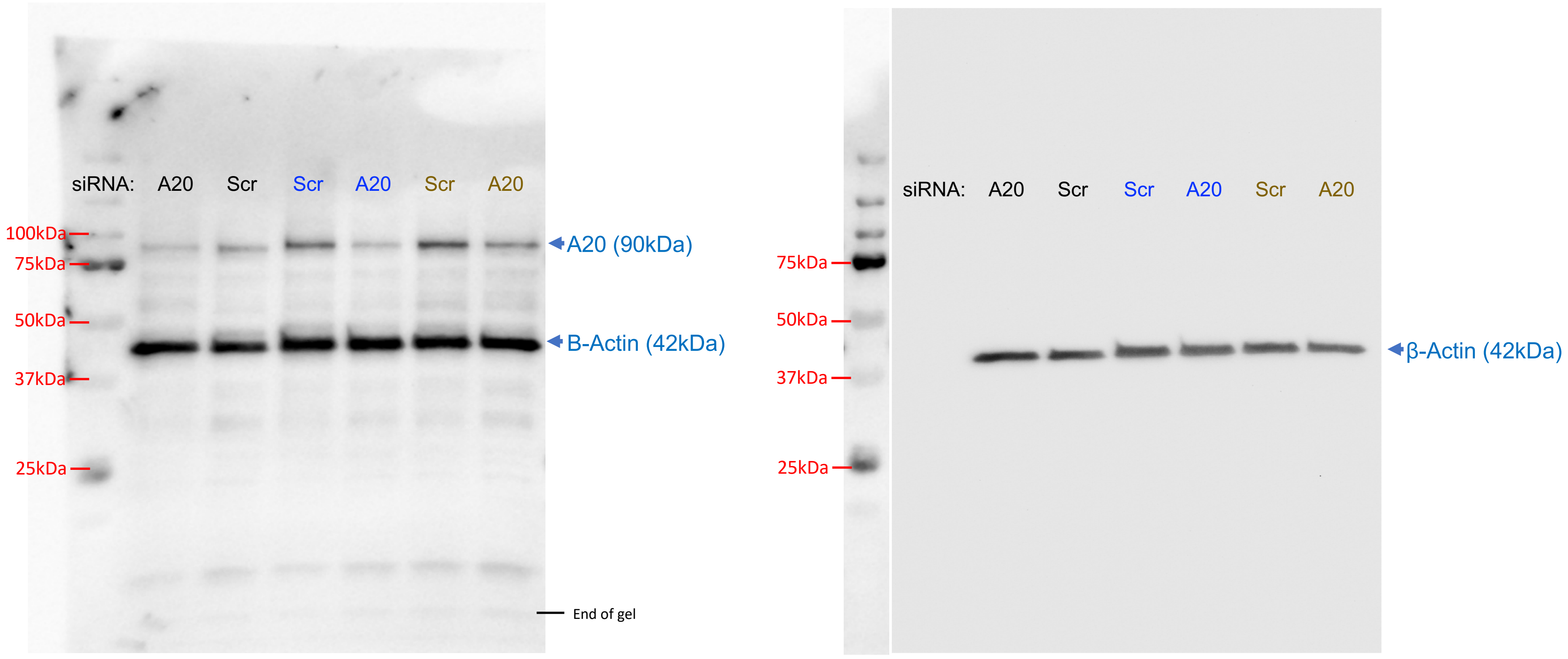

Membrane was probed first with β-actin followed by A20.
Bands were visualized using chemiluminescence (Amersham ECL Prime Western Blotting Detection Reagent) on a FOTO/Analyst® LuminaryFX Workstation.
Bands were quantified using ImageJ.

Notes:

1) As β-Actin signal is stronger than LMP7, we used the β-Actin image from the right panel (not saturated) for Figure 7a.
2) The A20 and β-Actin bands of the **Scr and A20 siRNA for the subject in black font (Subject 1) was flipped** and placed at the end to preserve the order of the membrane as depicted in the original Figure 7a. In revised Figure 7a, we now present the image as we captured without flipping the order of LMP7 or β-actin bands from cells of Subject 1.

Revised Fig 7a

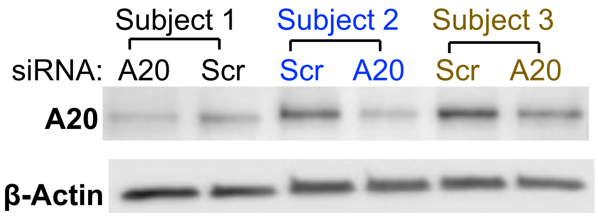

Figure 8. LMP7 Western blot

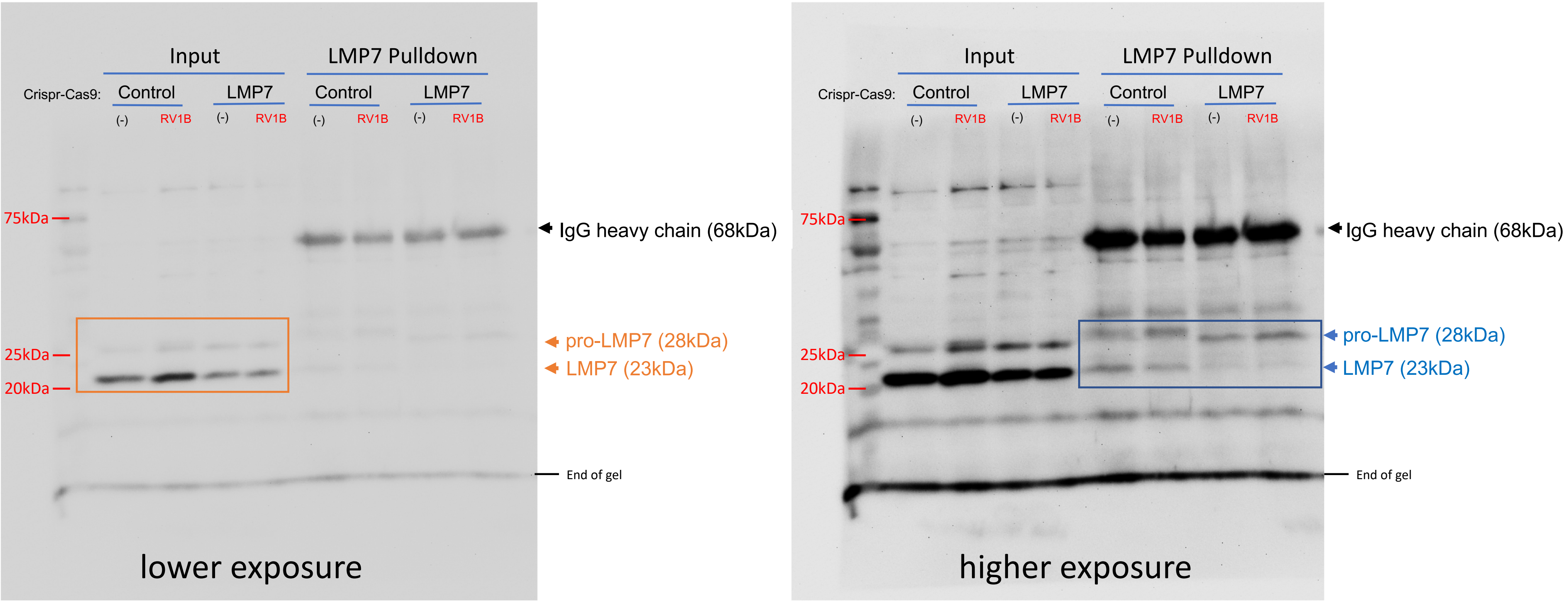

Membrane was probed first with LMP7 followed by A20 and then β-actin.
Bands were visualized using chemiluminescence (Amersham ECL Prime Western Blotting Detection Reagent) on a FOTO/Analyst® LuminaryFX Workstation

Figure 8. A20 and β-actin western blot

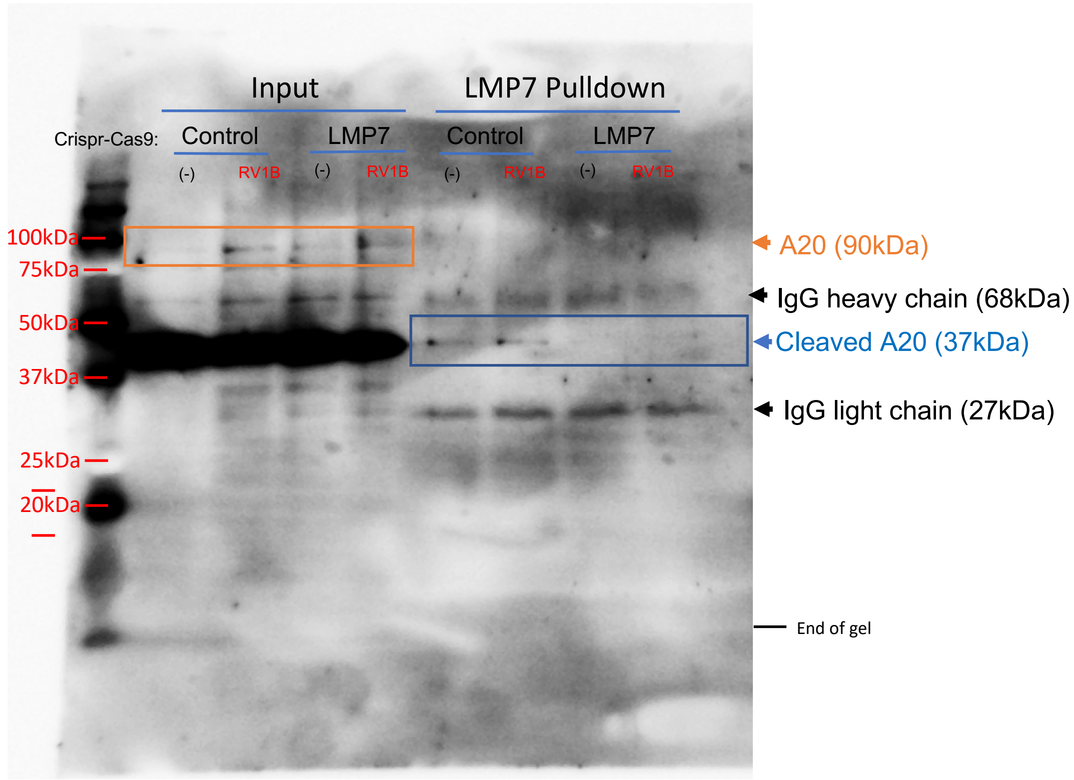

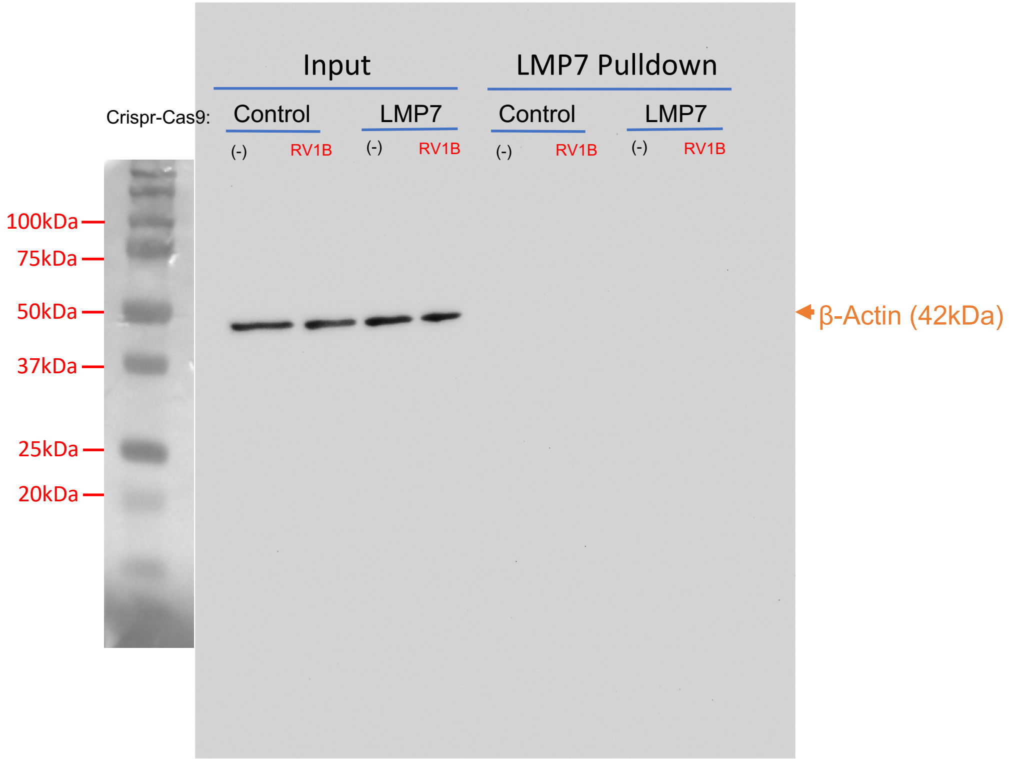

Membrane was probed first with LMP7 followed by A20 and then β-actin.
Bands were visualized using chemiluminescence (Amersham ECL Prime Western Blotting Detection Reagent) on a FOTO/Analyst® LuminaryFX Workstation.
